# Spi-C positively regulates RANKL-mediated osteoclast differentiation and function

**DOI:** 10.1038/s12276-020-0427-8

**Published:** 2020-04-27

**Authors:** Eun Mi Go, Ju Hee Oh, Jin Hee Park, Soo Young Lee, Na Kyung Lee

**Affiliations:** 1 Department of Medical Science, College of Medical Sciences, Soonchunhyang University, Asan, 31538 Korea; 2 Department of Life Science, The Research Center for Cellular Homeostasis, Ewha Womans University, Seoul, 03760 Korea

**Keywords:** Bone, Cell signalling

## Abstract

Spi-C is an SPI-group erythroblast transformation-specific domain transcription factor expressed during B-cell development. Here, we report that Spi-C is a novel receptor activator of nuclear factor-κB ligand (RANKL)-inducible protein that positively regulates RANKL-mediated osteoclast differentiation and function. Knockdown of Spi-C decreased the expression of RANKL-induced nuclear factor of activated T-cells, cytoplasmic 1, receptor activator of nuclear factor-κB (RANK), and tartrate-resistant acid phosphatase (TRAP), resulting in a marked decrease in the number of TRAP-positive multinucleated cells. Spi-C-transduced bone marrow-derived monocytes/macrophages (BMMs) displayed a significant increase in osteoclast formation in the presence of RANKL. In addition, Spi-C-depleted cells failed to show actin ring formation or bone resorption owing to a marked reduction in the expression of RANKL-mediated dendritic cell-specific transmembrane protein and the d2 isoform of vacuolar (H+) ATPase V0 domain, which are known osteoclast fusion-related genes. Interestingly, RANKL stimulation induced the translocation of Spi-C from the cytoplasm into the nucleus during osteoclastogenesis, which was specifically blocked by inhibitors of p38 mitogen-activated protein kinase (MAPK) or PI3 kinase. Moreover, Spi-C depletion prevented RANKL-induced MAPK activation and the degradation of inhibitor of κB-α (IκBα) in BMMs. Collectively, these results suggest that Spi-C is a novel positive regulator that promotes both osteoclast differentiation and function.

## Bone maintenance: regulating cells that break down bone

A gene-controlling protein called Spi-C promotes the development of bone-processing cells called osteoclasts; details of the molecular mechanisms involved will aid understanding of Spi-C’s role in bone health and disease. Osteoclasts degrade bone during the normal process of bone remodeling, balanced by the activity of osteoblast cells that form new bone. Excessive osteoclast activity can cause the bone loss associated with various bone diseases including early-onset osteoporosis. Researchers in South Korea led by Soo Young Lee at Ewha Womans University and Na Kyung Lee at Soonchunhyang University, Asan, found that Spi-C promotes osteoclast development by activating genes that code for key proteins of a signaling pathway known to be crucial for bone health. Drugs that interfere with Spi-C activity may therefore offer a new approach for treating bone disease.

## Introduction

Osteoclasts are multinucleated giant cells that can resorb bone. They induce skeletal development and continuous bone remodeling together with bone-forming osteoblasts^1,2^. Osteoclast precursors are derived from hematopoietic progenitors of the monocyte/macrophage lineage. Receptor activator of nuclear factor-κB ligand (RANKL) and macrophage colony-stimulating factor (M-CSF) are essential for osteoclast differentiation^3,4^. Excess osteoclast activity results in bone loss, as observed in various bone-related diseases, including postmenopausal osteoporosis^5–7^. Thus, studies of the regulatory mechanisms of osteoclast differentiation and function are necessary for the treatment and prevention of bone-related diseases.

The binding of RANKL to receptor activator of nuclear factor-κB (RANK) recruits tumor necrosis factor receptor-associated factor 6 and stimulates the downstream activation of extracellular signal-regulated kinase 1/2 (ERK1/2), c-Jun N-terminal kinase (JNK) 1/2, and p38 mitogen-activated protein kinases (MAPKs) and inhibitor of κB-α (IκBα) pathways^7,8^. The activation of these MAPKs and IκBα ultimately results in changes in the expression of various osteoclast marker genes, including nuclear factor of activated T cells, cytoplasmic 1 (NFATc1); tartrate-resistant acid phosphatase (TRAP); cathepsin K; and microphthalmia transcription factor^1,7,8^. NFATc1 is a master transcription factor that regulates osteoclastogenesis. Ectopic expression of constitutively active NFATc1 induces osteoclast formation, even in the absence of RANKL, whereas NFATc1-deficient embryonic stem cells fail to differentiate into osteoclasts^9^.

Mononuclear osteoclast precursors fuse to become mature multinucleated osteoclasts that form actin rings and degrade bone matrix^1,4,7^. Deficiency in the d2 isoform of vacuolar (H+) ATPase V0 domain (Atp6v0d2) or dendritic cell-specific transmembrane protein (DC-STAMP) results in osteopetrotic phenotypes due to defects in the cell–cell fusion process in mice, indicating that these genes are crucial regulators of osteoclastic cell–cell fusion^10,11^.

Complementary DNA microarrays have been utilized to identify molecules that play roles in osteoclast differentiation^12^. We found that Spi-C expression was increased during RANKL-induced osteoclastogenesis. Spi-C is an erythroblast transformation-specific domain transcription factor with a transactivation domain located at the N-terminus that is closely related to PU.1 and Spi-B^13–15^. Several transcription factors of the erythroblast transformation-specific protein family have been shown to be involved in gene expression, chromatin remodeling, cell cycle regulation, and differentiation^15–18^. Notably, Spi-C is known to be expressed during B-lymphocyte development and acts as both a positive and negative transcriptional regulator of B-lymphocyte differentiation^15^. However, the involvement of Spi-C in osteoclastogenesis remains unknown. To the best of our knowledge, this is the first study to report that Spi-C is a novel positive regulator of RANKL-induced osteoclast differentiation.

## Materials and methods

### Reagents and plasmids

Recombinant human M-CSF was purchased from R&D Systems (Minneapolis, MN, USA). RANKL was obtained from PeproTech EC Ltd. (London, England). Antibodies against Spi-C, NFATc1, β-actin, PU.1, c-Fms, TBP, tubulin, TRAP, and RANK were obtained from Santa Cruz Biotechnology Inc. Antibodies against phospho-ERK1/2, ERK1/2, phospho-JNK, JNK, phospho-p38, p38, and IκBα were purchased from Cell Signaling Technology (Beverly, MA, USA). Anti-Atp6v0d2 antibody, SB203580, PD98059, SP600125, SB216763, and LY294002 were obtained from Sigma-Aldrich (St. Louis, MO, USA). The pGFP-C-shLenti-Spi-C and pLenti-C-Myc-DDK-Spi-C vectors were purchased from OriGene Technologies Inc. (Rockville, MD, USA).

### Isolation of bone marrow precursors, cell culture, and in vitro osteoclastogenesis

The isolation of bone marrow precursors and the in vitro osteoclastogenesis experiments were performed as described previously^19^. In brief, bone marrow cells were isolated from the femurs of 4–6 week-old C57BL/6 male mice and incubated in α-MEM containing 10% fetal bovine serum and M-CSF (10 ng/ml). After 24 h, the nonadherent cells were harvested and cultured in α-MEM containing 10% fetal bovine serum and M-CSF (20 ng/ml) for 3 days. After washing out the nonadherent cells, the adherent cells were used as bone marrow-derived monocytes/macrophages (BMMs). For osteoclast formation, isolated BMMs were further incubated with 200 ng/ml RANKL and 30 ng/ml M-CSF. After 5 days, these cells were fixed and stained for TRAP using a TRAP staining kit (Sigma-Aldrich). The number of pink-colored TRAP-positive (TRAP+) multinucleated cells (MNCs; >3 nuclei) or large TRAP+ MNCs with >20 nuclei were counted and are presented as relative percentages. The cells were observed using a Zeiss Axiovert 200 microscope, and images were obtained with an AxioCam HR microscope camera (Carl Zeiss, Göttingen, Germany) equipped with Axio Vision 3.1 software (Carl Zeiss). RAW264.7 cells were cultured in DMEM containing 10% fetal bovine serum.

### Microarray experiments

Total RNA was isolated from BMMs treated with 200 ng/ml RANKL for 12 h or 24 h in the presence of M-CSF (30 ng/ml) and subjected to microarray experiments as previously described^12,20^. The raw data were extracted using Illumina GenomeStudio v2009.2 and Gene Expression Module v1.5.4 (Illumina, San Diego, CA, USA). The probe signal values were logarithmically transformed and normalized with the quantile method. Comparative analyses between the test and control groups were then performed using fold-change with the local-pooled-error test for the adjusted false discovery rate *p* value. False discovery was controlled by adjusting the *p* value using the Benjamini–Hochberg algorithm.

### RNA isolation and real-time polymerase chain reaction (qPCR)

Total RNA was isolated from BMMs treated with M-CSF (30 ng/ml) and/or RANKL (200 ng/ml) and reverse transcribed using SuperScript III reverse transcriptase (Invitrogen, Carlsbad, CA, USA) in accordance with the manufacturer’s protocol. Specific primers for genes and *hprt* (internal control) were purchased from Qiagen GmbH (Hilden, Germany). qPCR was performed in triplicate with Brilliant III Ultra-Fast SYBR Green qPCR Master Mix (Agilent Technologies, Santa Clara, USA) using an Mx3000P qPCR system (Agilent Technologies). The thermal cycling conditions were 3 min at 95°C, followed by 40 cycles of 95 °C for 10 s and 60 °C for 20 s and one cycle of 95 °C for 1 min, 55 °C for 30 s, and 95 °C for 30 s. All quantitative results were normalized to *hprt* expression^19^.

### Western blot analysis

BMMs or RAW264.7 cells were stimulated as indicated and lysed in lysis buffer (20 mM Tris-HCl [pH 7.5], 150 mM NaCl, 1 mM Na_2_EDTA, 1 mM EGTA, 1% Triton X-100, 2.5 mM sodium pyrophosphate, 1 mM beta-glycerophosphate, 1 mM Na_3_VO_4_, 1 μg/ml leupeptin, and 1 mM phenylmethylsulfonylfluoride). The supernatants were prepared after centrifugation. Then, the samples were subjected to electrophoresis on a sodium dodecyl sulfate-polyacrylamide gel and blotted onto a polyvinylidene difluoride membrane. Immunoblotting was performed using specific primary antibodies followed by incubation with horseradish peroxidase-conjugated secondary antibodies. The blots were then enhanced using an ECL western blotting Detection kit (Amersham Biosciences, GE Healthcare, Pittsburgh, PA, USA).

### Immunocytochemistry

BMMs were seeded into 96-well plates at a density of 3 × 10^4^ cells/well. After incubation with RANKL (200 ng/ml) and/or inhibitors, the cells were fixed with 4% paraformaldehyde for 5 min. After three rinses with phosphate-buffered saline (PBS), the cells were permeabilized in cold 0.2% Triton X-100 for 5 min and incubated with 5% bovine serum albumin/PBS for 30 min. Then, the cells were incubated with an antibody against Spi-C (diluted 1:50) for 2 h at room temperature. After washing three times with PBS, the cells were incubated with fluorescein isothiocyanate-conjugated goat anti-rabbit secondary antibody (diluted 1:200; Thermo Fisher Scientific, Rockford, IL, USA) for 1 h in the dark. Then, the nuclei were stained with 4’, 6-diamidino-2-phenylindole (DAPI; diluted 1:1000) for 30 min. After washing with PBS, the cells were observed and imaged using an Olympus CKX41 fluorescence microscope (Olympus, Tokyo, Japan).

### Small interfering RNA (siRNA) transfection and lentiviral-mediated gene transduction

The siRNA targeting mouse Spi-C and a scrambled nontargeting siRNA (negative control) were purchased from OriGene Technologies. The isolated BMMs were transfected with these siRNAs using FuGENE6 transfection reagent (Promega Corporation, Fitchburg, WI, USA) according to the manufacturer’s protocol. After 24 h, the BMMs were used for osteoclast formation, gene expression, and protein analyses. The lentiviral packaging was performed using a lentiviral packaging system (OriGene Technologies). Briefly, HEK293T cells were transfected with premixed packaging plasmids and Spi-C shRNA lentiviral plasmids or Spi-C-expressing lentiviral plasmids using polyethylenimine (Sigma-Aldrich, St. Louis, MO, USA). The supernatants were collected 48 h after transfection, filtered, and used as viral stocks. For lentiviral infection, BMMs were incubated with the lentivirus stock and polybrene (10 μg/ml) for 6 h. The cells were used for western blot analysis or in vitro assays for osteoclast formation 2 days after infection.

### Actin ring formation assay

BMMs or mature osteoclasts were infected with the Spi-C shRNA lentivirus stock and polybrene (10 μg/ml) for 24 h. After 48 h, the BMMs were treated with 200 ng/ml of RANKL for 4 days. The BMMs and the mature osteoclasts were fixed with 4% formaldehyde, permeabilized with 0.1% Triton X-100, and incubated with Alexa Fluor 488-phalloidin (Invitrogen) for 20 min. Finally, the nuclei were stained with DAPI for 20 min. After washing with PBS, the cells were photographed using fluorescence microscopy.

### Bone resorption assay

BMMs or mature osteoclasts on dentine discs (Immunodiagnostic Systems Holdings Plc, Boldon Colliery, UK) were infected with the Spi-C shRNA lentivirus stock and polybrene (10 μg/ml) for 24 h. After 48 h, the BMMs were treated with 200 ng/ml of RANKL for 4 days. Finally, the BMMs and the mature osteoclasts were completely removed from the dentine discs, and the dentine discs were stained with hematoxylin. The resorption pits were visualized under a light microscope and measured and are presented as the relative pit area (%).

### Cytosolic and nuclear fractionation

RAW264.7 cells were treated with 200 ng/ml RANKL for 60 min and then lysed with cytosolic extraction buffer (10 mM HEPES [pH 7.4], 10 mM KCl, 1.5 mM MgCl_2_, 0.5 M dithiothreitol, and 0.05% NP-40) containing protease inhibitors and phosphatase inhibitors. After centrifugation, the supernatants were collected for use as cytosolic fractions. Then, the pellets were washed with cytosol extraction buffer and lysed with nuclear extraction buffer (5 mM HEPES [pH 7.4], 300 mM NaCl, 1.5 mM MgCl_2_, 0.2 mM EDTA, and 25% glycerol) containing protease inhibitors and phosphatase inhibitors. After incubation on ice for 30 min, the nuclear fraction was collected following centrifugation at 12,000 × *g* for 30 min at 4 °C.

### Statistical analysis

The results are presented as means ± standard deviation (SD) of at least three independent experiments. Statistical analyses were performed using Student’s *t* tests. A *p* value of <0.05 was considered statistically significant.

## Results

### Spi-C is upregulated during RANKL-mediated osteoclast differentiation

**Fig. 1:**
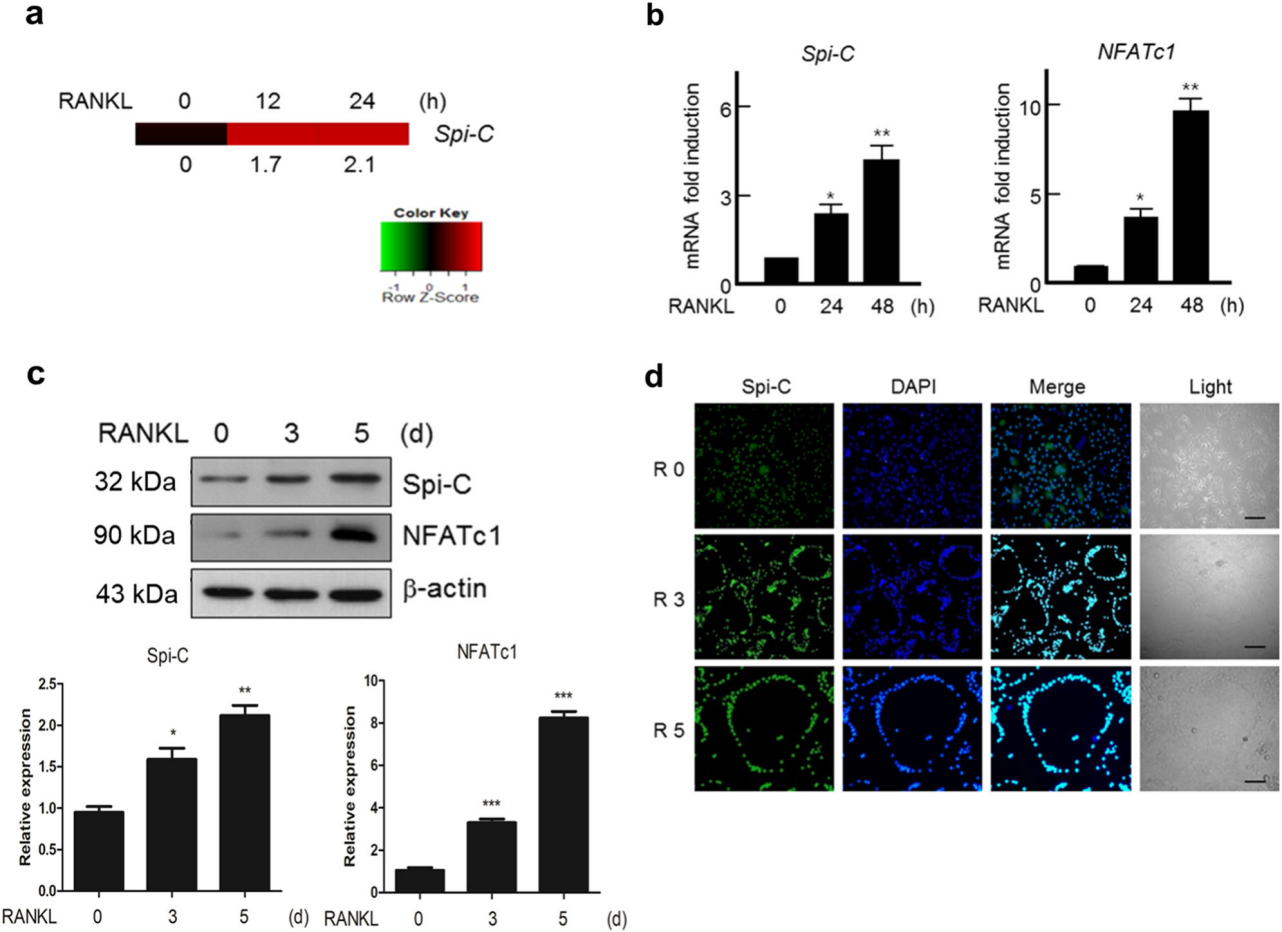
Spi-C is upregulated during RANKL-mediated osteoclast differentiation. **a** Gene chip analysis result of the *Spi-C* gene in BMMs that were stimulated with RANKL for 12 h and 24 h. **b** qPCR analysis of the mRNA expression levels of *Spi-C* and *NFATc1* in RANKL-stimulated BMMs at the indicated times. All quantitations were normalized to *hprt* expression. **c** Isolated BMMs were stimulated with RANKL (200 ng/ml) for the indicated times followed by lysis and western blot analysis with antibodies specific to Spi-C, NFATc1, and β-actin (upper panels). The protein bands were quantified using densitometry, and the levels of Spi-C and NFATc1 were normalized to the levels of actin. The relative expression levels in the stimulated cells compared with the untreated cells are shown (lower panels). The data are presented as the mean ± SD of three independent experiments. *p < 0.05, **p < 0.005 vs. nontreated cells. **d** BMMs stimulated with RANKL (200 ng/ml) for the indicated times were fixed and then subjected to immunofluorescence staining for Spi-C. Spi-C was detected as green fluorescence using FITC-conjugated secondary antibodies. The nuclei were stained blue with DAPI. These cells were observed and imaged under a fluorescence microscope. Scale bars, 100 μm (original magnification, ×100).

Microarray analysis was performed^12^ to investigate the molecules that are expressed during RANKL-induced differentiation of BMMs into osteoclasts and are potentially involved in osteoclastogenesis. Spi-C expression levels were increased by RANKL stimulation for 12 h and 24 h (Fig. 1a). The induction of the RANKL-mediated Spi-C gene was confirmed by qPCR analysis. RANKL treatment for 24 h or 48 h upregulated the expression of Spi-C (Fig. 1b). The same was true in response to RANKL treatment for 3 or 5 days, in which RANKL stimulation notably induced the expression of Spi-C in a time-dependent manner (Fig. 1c). Spi-C expression was not detected in osteoblasts (Supplementary Fig. S1), suggesting that Spi-C has a role during osteoclast formation. Furthermore, we observed the localization of Spi-C during RANKL-induced osteoclastogenesis. In the absence of RANKL, Spi-C localized to both the cytoplasm and the nucleus in BMMs (Fig. 1d). However, Spi-C was more clearly localized in the nucleus in RANKL-induced multinucleated osteoclasts (Fig. 1d). Interestingly, M-CSF alone induced Spi-C expression in BMMs (Supplementary Fig. S2a, b).

### Spi-C promotes RANKL-mediated osteoclast differentiation

**Fig. 2:**
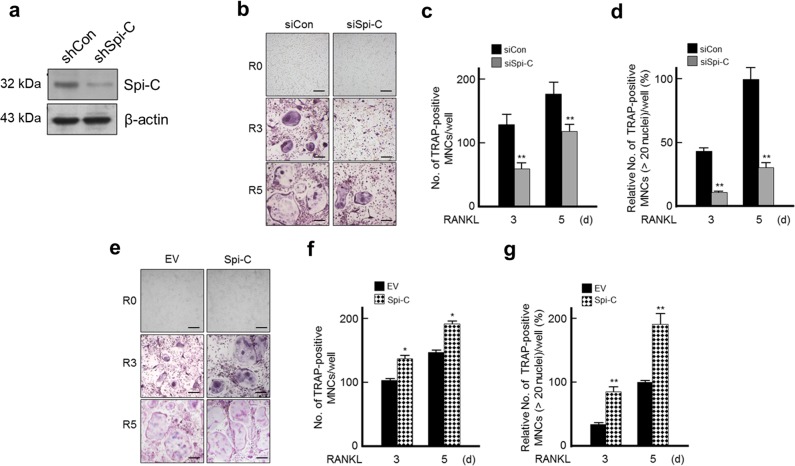
Spi-C promoted RANKL-mediated osteoclast differentiation. **a** After transfecting BMMs with a scrambled nontargeting siRNA (siCon) or Spi-C siRNA (siSpi-C), the cells were incubated with RANKL (200 ng/ml) for 3 days. Then, the cells were lysed and subjected to western blot analysis with the indicated antibodies. **b**–**d** BMMs transfected with a scrambled nontargeting siRNA (siCon) or Spi-C siRNA (siSpi-C) were cultured with RANKL (200 ng/ml) for the indicated times. TRAP staining was performed (original magnification, ×100) **b**, and the number of TRAP+MNCs (>3 nuclei) **c** or large TRAP+MNCs with >20 nuclei **d** were counted and are presented as relative percentages (%). **e**–**g** BMMs infected with an empty lentiviral vector (EV) or a Spi-C-expressing lentiviral vector (Spi-C) were cultured with RANKL (200 ng/ml) for the indicated times. Then, TRAP staining was performed as described in Fig. 2b. Scale bars, 100 μm **b**, **e**. The data are presented as the mean ± SD of three independent experiments. *p < 0.05, **p < 0.005.

The increase in Spi-C expression and the change in its localization during osteoclast formation led us to examine whether Spi-C regulates osteoclast differentiation. Spi-C siRNA effectively depleted the expression of Spi-C in BMMs (Fig. 2a). Knockdown of the *Spi-C* gene resulted in a marked decrease in the number of TRAP+MNCs compared with that of the controls (Fig. 2b, c). The formation of large TRAP+MNCs with >20 nuclei was significantly blocked by Spi-C depletion (Fig. 2d). To further confirm the role of Spi-C in osteoclast differentiation, we overexpressed Spi-C in BMMs using a Spi-C-expressing lentivirus. In the presence of RANKL, Spi-C-transduced BMMs displayed increased osteoclast formation compared with that of the controls (Fig. 2e, f). Consistently, the formation of large TRAP+MNCs with >20 nuclei was promoted by Spi-C overexpression (Fig. 2g).

### Spi-C regulates osteoclast marker gene expression

**Fig. 3:**
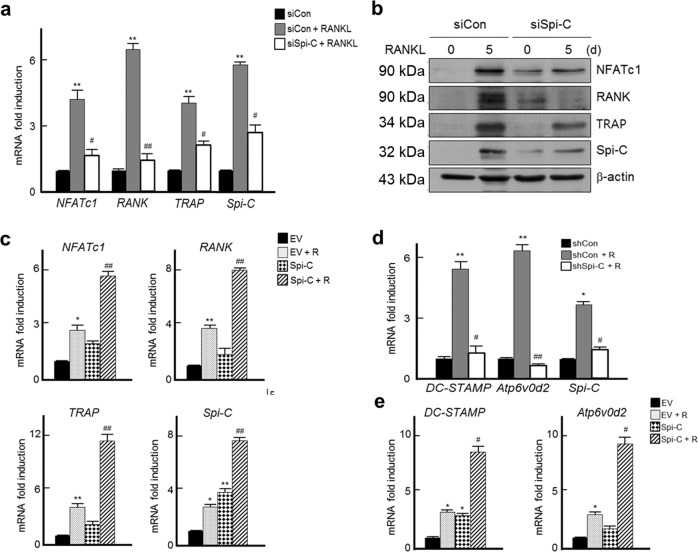
Spi-C regulates osteoclast marker gene expression. **a**, **b** BMMs transfected with a scrambled nontargeting siRNA (siCon) or Spi-C siRNA (siSpi-C) were cultured without or with RANKL for 24 h **a** or 5 days **b**. Then, qPCR **a** or western blot analysis **b** was performed. **c** BMMs infected with an empty lentiviral vector (EV) or Spi-C-expressing lentiviral vector (Spi-C) were cultured without or with RANKL (200 ng/ml) for 24 h, and qPCR was performed. **d** Isolated BMMs infected with control shRNA lentiviral vector (shCon) or Spi-C shRNA expression lentiviral vector (shSpi-C) were cultured without or with RANKL (200 ng/ml) for 48 h, and qPCR was performed. **e** BMMs infected with empty lentiviral vector (EV) or Spi-C-expressing lentiviral vector (Spi-C) were cultured without or with RANKL (200 ng/ml) for 48 h, and qPCR was performed. The data are presented as the mean ± SD of three independent experiments. *p < 0.05, **p < 0.005 vs. untreated cells and ^#^*p* < 0.05, ^##^*p* < 0.005 vs. RANKL-treated cells.

We next examined whether Spi-C regulates osteoclast marker gene expression. Knockdown of Spi-C decreased the expression of RANKL-induced *NFATc1*, *RANK*, and *TRAP* genes (Fig. 3a). The same results were observed when the protein levels in the cells were analyzed (Fig. 3b). Notably, when BMMs were infected with empty or Spi-C-expressing lentivirus, Spi-C overexpression slightly induced osteoclast marker gene expression, even in the absence of RANKL (Fig. 3c). Moreover, Spi-C overexpression in the presence of RANKL significantly enhanced the expression of osteoclast markers, including *NFATc1*, *RANK*, and *TRAP* (Fig. 3c). To further confirm whether Spi-C regulates *RANK, NFATc1*, and *TRAP* induction at the transcriptional level, we examined the recruitment of Spi-C to the *NFATc1*, *RANK*, and *TRAP* promoters following RANKL stimulation in BMMs using chromatin immunoprecipitation assays. The results revealed increased Spi-C recruitment to the *NFATc1* and *RANK* promoters but not to the *TRAP* promoter after 1 day of RANKL stimulation (Supplementary Fig. S3).

As Spi-C overexpression significantly increased the formation of large TRAP+MNCs (Fig. 2e, g), we further investigated whether Spi-C regulates the expression of the *DC-STAMP* and *Atp6v0d2* genes, which are known to be related to osteoclast fusion. RANKL stimulation markedly increased the expression of both genes. However, Spi-C depletion by Spi-C shRNA-expressing lentivirus markedly blocked RANKL-mediated expression of *DC-STAMP* and *Atp6v0d2* (Fig. 3d). In contrast, RANKL-induced expression of *DC-STAMP* and *Atp6v0d2* was significantly enhanced by Spi-C overexpression (Fig. 3e). Even without RANKL, Spi-C-transduced BMMs displayed increased expression of *DC-STAMP* and *Atp6v0d2*, consistent with the results of previously mentioned osteoclast marker genes (Fig. 3c). These results suggest that Spi-C has a role as a positive regulator of osteoclast formation by increasing the expression of osteoclast marker genes.

### Spi-C regulates actin ring formation and bone resorption by osteoclasts

**Fig. 4:**
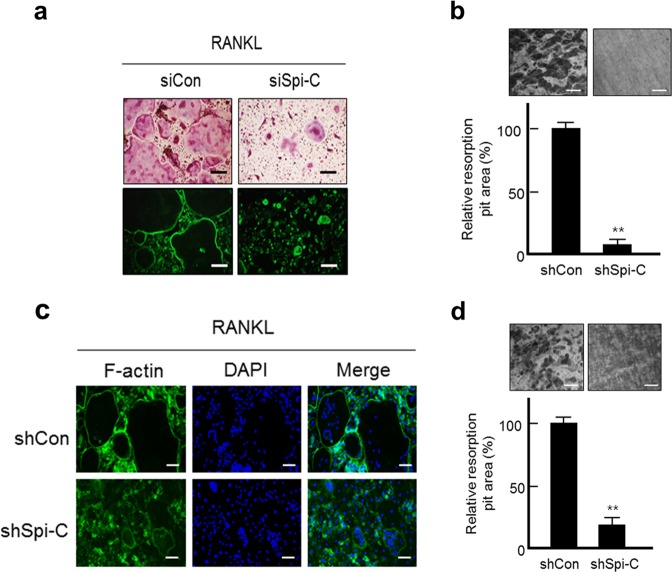
Spi-C promotes RANKL-induced actin ring formation and bone resorption. **a** BMMs transfected with a scrambled nontargeting siRNA (siCon) or Spi-C siRNA (siSpi-C) were cultured with RANKL (200 ng/ml) for 5 days. Then, the cells were fixed and subjected to TRAP staining (upper panel) or immunofluorescence staining for F-actin (lower panel). Scale bars, 100 μm (original magnification, ×200). **b**, **d** BMMs **b** or mature osteoclasts **d** on dentine discs were infected with the control shRNA lentiviral vector (shCon) or Spi-C shRNA expression lentiviral vector (shSpi-C) for 24 h. After 48 h, the BMMs or mature osteoclasts were treated with 200 ng/ml of RANKL for 4 days **b** or 2 days **d**. The BMMs and the mature osteoclasts were completely removed from the dentine discs. Then, the dentine discs were stained with hematoxylin, and the resorption pits were visualized under a light microscope (upper panel). Scale bars, 200 μm (original magnification, ×200). The resorption pit areas were measured and are presented as the relative pit area (%). **c** Mature osteoclasts were infected with a control shRNA lentiviral vector (shCon) or Spi-C shRNA expression lentiviral vector (shSpi-C) for 24 h. After 48 h, the cells were subjected to immunofluorescence staining for F-actin. Nuclei stained with DAPI were observed as blue and imaged under a fluorescence microscope. Scale bars, 100 μm (original magnification, ×200). The data are presented as the mean ± SD of three independent experiments. **p < 0.005 vs. untreated cells.

Mature multinucleated osteoclasts have a sealing zone that is required for bone resorption^1,4,7^. Thus, we determined whether actin ring formation and bone resorption were affected by Spi-C depletion. Spi-C-depleted BMMs failed to form actin rings with clearer and denser margins than the control cells (Fig. 4a). Likewise, the area of bone resorption pits was dramatically decreased by knockdown of Spi-C compared with that of the control cells (Fig. 4b). As the reduction in actin ring formation and bone resorption was probably caused by decreased osteoclast formation owing to Spi-C depletion, we used mature osteoclasts instead of BMMs to verify the effect of Spi-C. The results were similar, even when Spi-C was depleted in mature osteoclasts (Fig. 4c, d). These findings indicate that Spi-C has a critical role in actin ring formation and the bone-resorbing activity of mature osteoclasts.

### Nuclear translocation of Spi-C is regulated through p38 and PI3 kinase (PI3K)

**Fig. 5:**
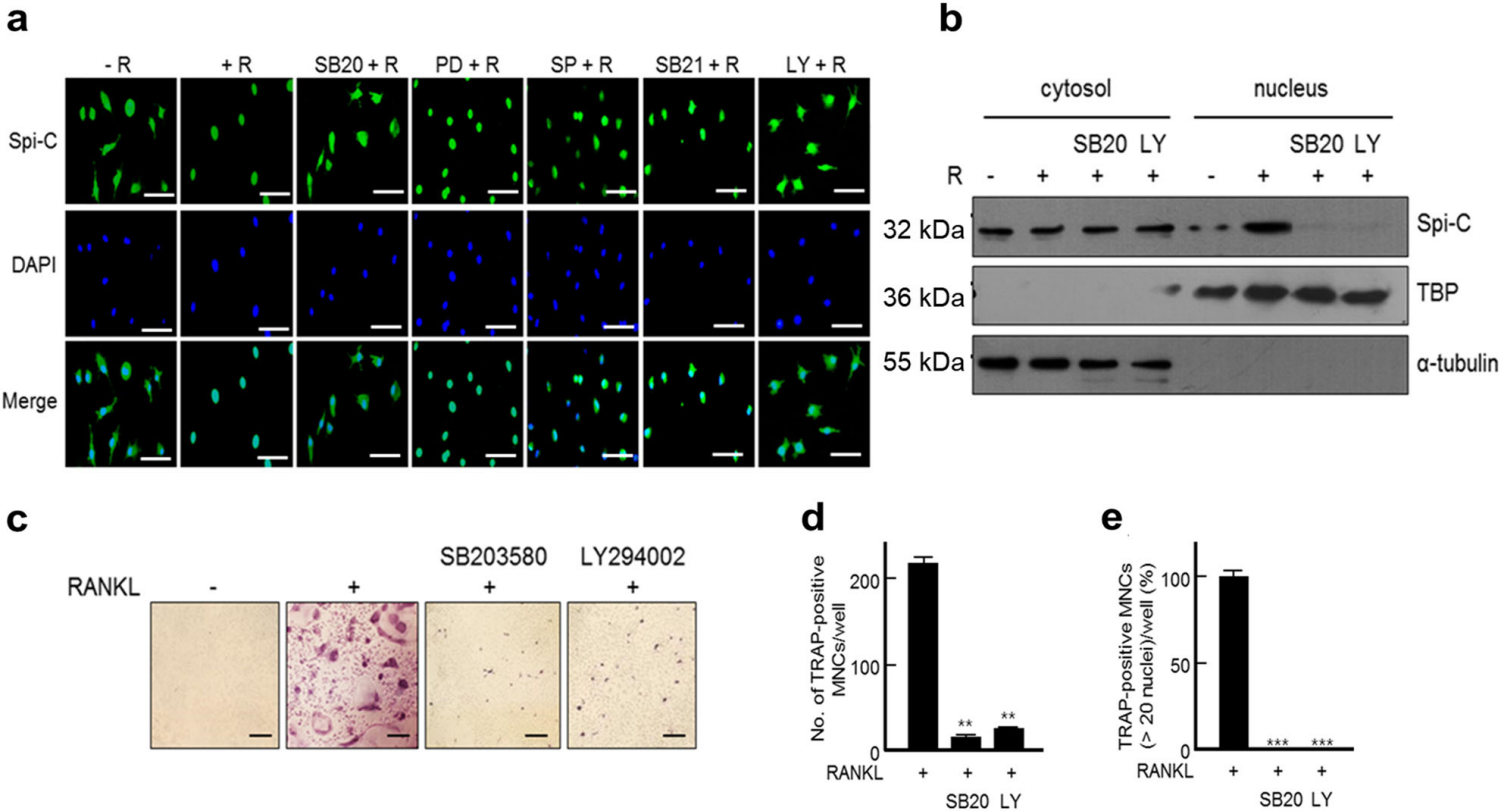
Nuclear translocation of Spi-C through p38 and PI3K. **a** BMMs that had been serum starved for 6 h were pretreated with the inhibitors SB203580 (SB20, 20 μM), PD98059 (PD, 20 μM), SP600125 (SP, 10 μM), SB216763 (SB21, 100 nM), and LY294002 (LY, 5 μM) for 30 min and then incubated with RANKL (200 ng/ml) for 90 min. Then, the cells were subjected to immunofluorescence staining for Spi-C. The nuclei were stained blue with DAPI. The cells were observed and imaged under a fluorescence microscope. Scale bars, 100 μm (original magnification, ×400). **b** RAW264.7 cells were treated with RANKL (200 ng/ml) for 2 days to induce Spi-C expression and serum starved for 6 h. The cells were pretreated with SB203580 (SB20, 20 μM) or LY294002(LY, 5 μM) for 30 min and then incubated with RANKL (50 ng/ml) for 60 min. Then, the cells were subjected to nuclear fractionation. Tubulin and TBP were used as markers for the cytosol and nucleus, respectively. **c**–**e** The isolated BMMs were pretreated with SB203580 (SB20, 20 μM) or LY294002 (LY, 5 μM) for 30 min and then incubated with RANKL (200 ng/ml) for 5 days. Thereafter, TRAP staining was performed (original magnification, ×100) (**c**), and the number of TRAP+MNCs (>3 nuclei) **d** or large TRAP+multinucleated osteoclasts with >20 nuclei **e** were counted and are presented as relative percentages (%). Scale bars, 100 μM **c**. The data are presented as the mean ± SD of three independent experiments. **p < 0.005, ***p < 0.001 vs. RANKL-treated cells.

As Spi-C translocated into the nucleus during RANKL-induced osteoclast differentiation (Fig. 1d), we investigated the RANKL-mediated signaling pathway that affects the nuclear translocation of Spi-C. RANKL stimulation induced the translocation of Spi-C from the cytoplasm into the nucleus, which was specifically blocked by pretreatment with SB203580 and LY294002, inhibitors of p38 MAPK and PI3K, respectively (Fig. 5a). In contrast, inhibitors of MAPKs or NF-κB did not affect Spi-C translocation. The cytosolic and nuclear fractionation assays supported the results described above, in which SB203580 and LY294002 pretreatment significantly decreased RANKL-stimulated nuclear translocation of Spi-C (Fig. 5b). We found that both SB203580 and LY294002 completely inhibited RANKL-induced osteoclast differentiation as well as the formation of large TRAP+MNCs with >20 nuclei (Fig. 5c–e). These results suggest that the RANKL-mediated p38 and PI3K signaling pathways are important for the nuclear translocation of Spi-C, which might induce osteoclast differentiation.

### Spi-C expression is regulated through the JNK signaling pathway

**Fig. 6:**
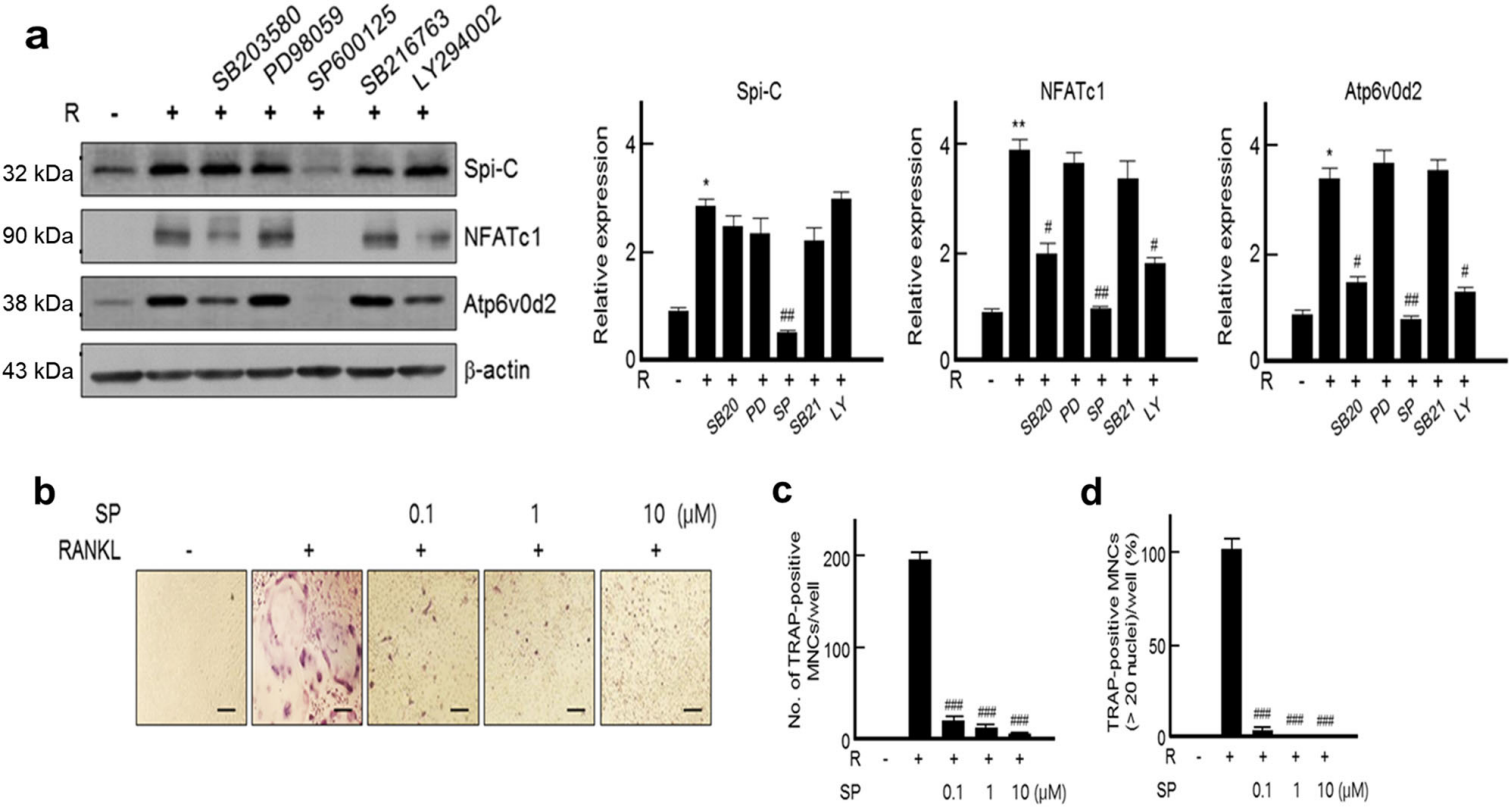
Regulation of Spi-C expression through the JNK signaling pathway. **a** Isolated BMMs were pretreated with the indicated inhibitors as described in Fig. 5a and incubated with RANKL (200 ng/ml) for 3 days. Then, the cells were lysed and subjected to western blot analysis. Actin was used as a loading control (left panel). The protein bands were quantified by densitometry, and the levels of Spi-C, NFATc1, and Atp6v0d2 were normalized to the levels of actin. The relative expression levels in the stimulated cells compared with the untreated cells are shown (right panels). The results are representative of at least three independent experiments. **b**–**d** Isolated BMMs were pretreated with the indicated concentrations of SP600125 (SP) for 30 min and incubated with RANKL (200 ng/ml) for 5 days. Then, TRAP staining was performed (original magnification, ×100) **b**, and the number of TRAP+MNCs (>3 nuclei) or large TRAP+multinucleated osteoclasts with >20 nuclei **d** were counted and are presented as relative percentages (%). Scale bars, 100 μm **b**. The data are presented as the mean ± SD of three independent experiments. *p < 0.05, **p < 0.005 vs. untreated cells and ^#^*p* < 0.05, ^##^*p* < 0.005, ^###^*p* < 0.001 vs. RANKL*-*treated cells.

We examined the signaling pathway of RANKL-induced Spi-C expression. RANKL-mediated Spi-C expression was only decreased by pretreatment with SP600125, a JNK inhibitor, whereas the other inhibitors failed to regulate Spi-C expression (Fig. 6a). Likewise, the expression of both NFATc1 and Atp6v0d2 was significantly inhibited by SP600125 pretreatment (Fig. 6a). Interestingly, their expression was also partially blocked by pretreatment with SB203580, which is a p38 inhibitor, or LY294002, which is a PI3K inhibitor (Fig. 6a). Moreover, SP600125 pretreatment prevented RANKL-induced osteoclast differentiation and the formation of larger multinucleated osteoclasts with >20 nuclei in a dose-dependent manner (Fig. 6b–d). These results indicate that Spi-C expression through the JNK signaling pathway is crucial for RANKL-mediated osteoclast differentiation.

### Spi-C depletion inhibits RANKL-mediated NF-κB and MAPK activation

**Fig. 7:**
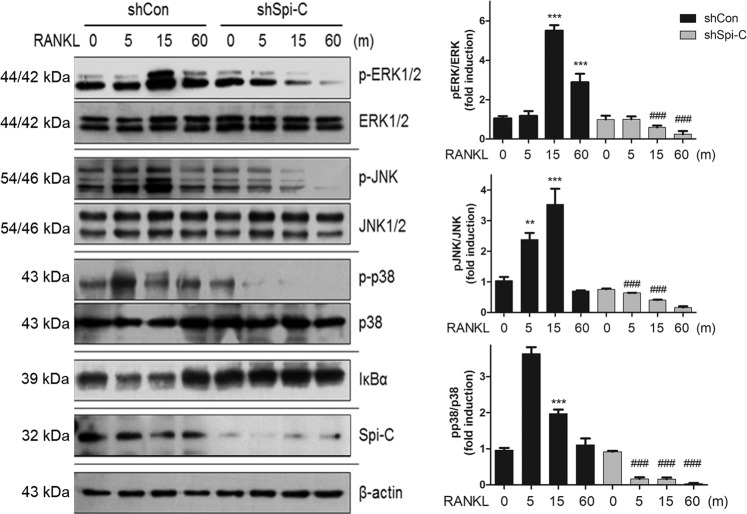
Decrease in RANKL-mediated NF-κB and MAPK activation by Spi-C depletion. BMMs were transduced with a control shRNA lentiviral vector (shCon) or Spi-C shRNA expression lentiviral vector (shSpi-C), serum-starved, and incubated with RANKL (200 ng/ml) for the indicated times. Then, the cells were subjected to western blot analysis with antibodies specific to p-ERK1/2, ERK1/2, p-JNK, JNK1/2, p-p38, p38, IκBα, Spi-C, and actin. Actin was used as a loading control (right panels). The protein bands were quantified using densitometry, and the levels of p-ERK1/2, p-JNK and p-p38 were normalized to the levels of ERK1/2, JNK1/2 and p38, respectively (left panels). **p < 0.005, ***p < 0.001 vs. untreated shCon cells and ^###^*p* < 0.001 vs. RANKL-treated shCon cells.

Given our data that Spi-C knockdown downregulated RANK receptor expression (Fig. 3a), we explored the effects of Spi-C on the activation of RANKL-dependent MAPK and NF-κB pathways. As expected, MAPKs, such as ERK1/2, JNK1/2, and p38 MAPK, were activated by RANKL stimulation. However, these responses were markedly attenuated in Spi-C-depleted BMMs (Fig. 7). Although IκBα was decreased by RANKL at 15 and 30 min, it was not decreased in Spi-C-depleted BMMs (Fig. 7). These data further suggest that Spi-C is involved in osteoclast formation by regulating RANK signaling.

## Discussion

The excessive bone destruction that is observed in many bone-related diseases is mainly caused by increased osteoclast activity^3–6^. Thus, many studies have been performed to uncover the differentiation mechanism of bone-resorbing osteoclasts^5–8^. We found that Spi-C expression was increased during osteoclast formation by RANKL stimulation^12,20^.

To date, little has been studied on the role of Spi-C in osteoclasts. Here, we suggest that Spi-C acts as a novel regulatory factor to promote RANKL-mediated osteoclast differentiation and bone-resorbing function.

Knockdown of Spi-C reduced RANKL-mediated osteoclast differentiation, whereas Spi-C transduction into BMMs showed the opposite effect. Although Spi-C transduction in the absence of RANKL increased the expression of osteoclast marker genes in BMMs, it was insufficient to induce osteoclast differentiation. Instead, Spi-C enhanced osteoclast formation via synergistic augmentation of the RANKL-induced expression of osteoclast marker genes, such as *NFATc1*, *RANK*, and *TRAP*, by RANKL. NFATc1, a key osteoclastogenic regulator, translocates into the nucleus where it induces numerous osteoclast-specific target genes responsible for cell fusion and function, such as *DC-STAMP* and *Atp6v0d2*, in the late stage of osteoclastogenesis^21–25^. *DC-STAMP*-deficient mice have been shown to exhibit moderate osteopetrosis, which was distinguished by TRAP+ mononuclear osteoclasts that could resorb bone inefficiently^26^, and mice that lacked *Atp6v0d2* were shown to exhibit mild osteopetrosis owing to the absence of multinuclear TRAP+ osteoclasts, although they possessed mononuclear TRAP+ cells^27^.

Our data suggest that Spi-C governs both early and late stages of osteoclast differentiation, as well as bone-resorbing functions. The discovery of RANKL/RANK and the elucidation of NFATc1 functions in osteoclasts has been central to understanding the mechanisms underlying osteoclast differentiation, cell fusion, and bone resorption^3,28^. We showed that Spi-C binds with *RANK* and *NFATc1* promoters and consequently increases RANK receptor and NFATc1 expression during osteoclast formation. Given the importance of RANK receptors and NFATc1 in osteoclasts, Spic-C may be involved in the entire process of osteoclast differentiation and function.

Our results showed that JNK activity regulates Spi-C expression and that Spi-C in turn regulates the activation of MAPKs. These results suggest that a positive feedback regulatory loop exists between Spi-C and MAPKs during osteoclast formation. We showed that Spi-C binds with the *RANK* promoter, which induces RANK expression. Consequently, the Spi-C-induced increase in RANK receptor expression leads to MAPK activation. Thus, our results suggested that Spi-C-dependent RANK upregulation is responsible for the association between Spi-C and MAPKs.

Spi-C deficiency caused a decrease in the expression of DC-STAMP and Atp6v0d2, leading to the failure of actin ring formation and a marked reduction in the bone-resorbing activity of osteoclasts. Because an increase in osteoclast activity can result in bone-related diseases^1,2^, such as postmenopausal osteoporosis, targeting the mechanism that controls osteoclast activity could be more effective than the current drugs used to treat such diseases.

**Fig. 8:**
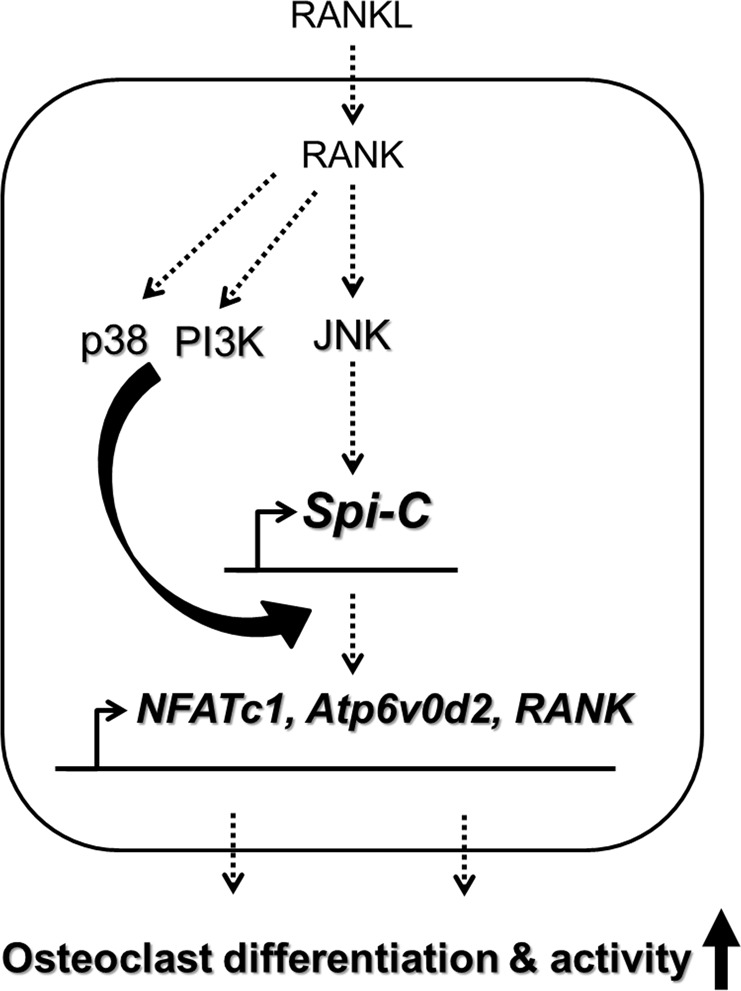
A proposed model for the role of Spi-C during RANKL-mediated osteoclast differentiation and function. Spi-C is a transcription factor induced by the RANKL-mediated JNK pathway. The activation of p38 MAPK and PI3K by RANKL regulates the nuclear translocation of Spi-C to induce osteoclast marker gene expression, thus increasing osteoclast formation and bone resorption function.

No studies have reported Spi-C functions in osteoclasts, either in vitro or in vivo. By identifying Spi-C as a novel transcriptional regulator of RANK signaling in osteoclasts, this study provides new insights regarding the mechanism for the transcriptional regulation of RANK signaling (Fig. 8). Notably, transcription factors that regulate osteoclast formation maintain bone development and metabolism under physiological conditions and regulate bone loss under pathological conditions, such as inflammatory states^29,30^. Further research regarding the pathophysiological roles played by Spi-C remains necessary to understand whether Spi-C acts as a key transcriptional regulator that promotes osteoclast formation. Such studies will provide us with a deeper understanding of bone homeostasis and help to expand insight into the development of therapeutic agents for various bone diseases.

## Supplementary information

Supplementary Information
