# Supplementary material for: Spi-C positively regulates RANKL-mediated osteoclast differentiation and function
Source: Exp Mol Med. 2020 Apr 27;52(4):691–701. doi: 10.1038/s12276-020-0427-8 (PMC7210314; doi:10.1038/s12276-020-0427-8)
Supplement: Supplementary file 1 — Supplementary Information [file 12276_2020_427_MOESM1_ESM.docx]

**Supplementary Information**

**Spi-C positively regulates RANKL-mediated osteoclast differentiation and function**

Running title: Role of Spi-C in osteoclasts

Eun Mi Go^1,*^, Ju Hee Oh^1,*^, Jin Hee Park^2^, Soo Young Lee^2,§^, and Na Kyung Lee^1,§^

^1^Department of Medical Science, College of Medical Sciences, Soonchunhyang University, Asan 31538, Korea

^2^Department of Life Science, The Research Center for Cellular Homeostasis, Ewha Womans University, Seoul 03760, Korea

^*^These authors contributed equally to this work.

^§^Corresponding author: Soo Young Lee, Department of Life Science, The Research Center for Cellular Homeostasis, Ewha Womans University, Seoul 03760, Korea. Tel: +82 2 3277 4257, E-mail: [leesy@ewha.ac.kr](mailto:leesy@ewha.ac.kr); Na Kyung Lee, Department of Medical Science, College of Medical Sciences, Soonchunhyang University, Asan 31358, Korea. Tel: +82 41 530 3036, E-mail: [nlee1116@hotmail.com](mailto:nlee1116@hotmail.com)


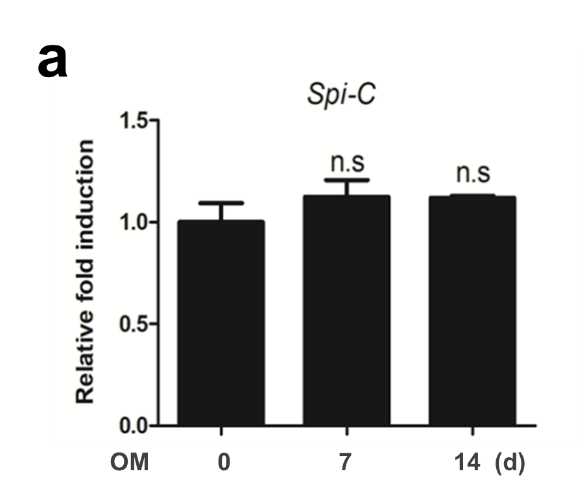


**Supplementary Figure S1. Spi-C expression in calvarial osteoblasts. a** qPCR results showing *Spi-C* mRNA expression levels in fetal mouse calvarial osteoblast cultures. All quantifications were normalized against *hprt* mRNA expression levels. Data are presented as the mean ± SD of three independent experiments. n.s, not significant; OM, osteogenic medium.

**
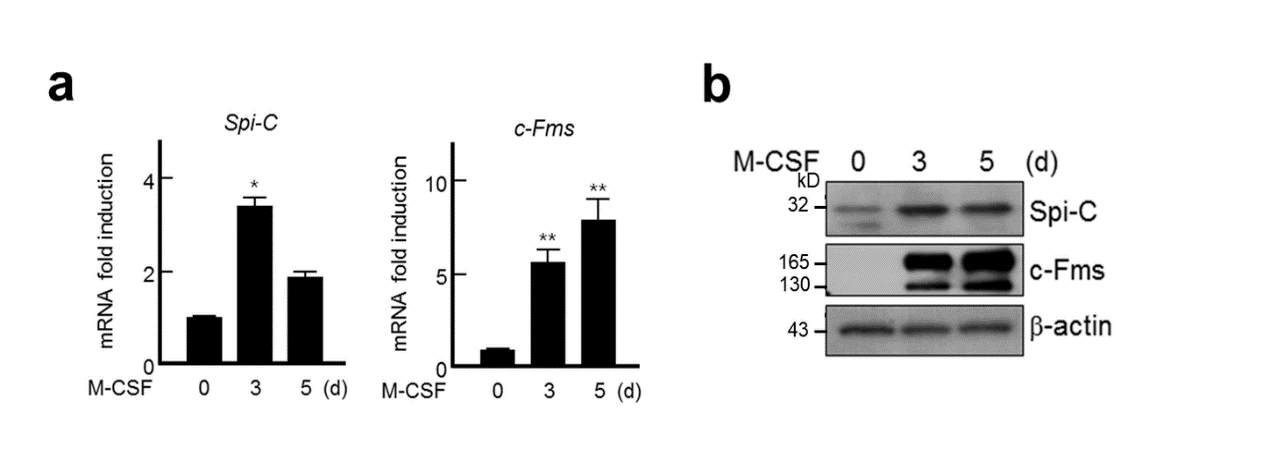
**

**Supplementary Figure S2. M-CSF induces *Spi-C* expression in BMMs**. **a** qPCR results showing the *Spi-C* and *c-Fms* mRNA expression levels in BMMs stimulated with M-CSF (30 ng/ml) for 3 and 5 days. All quantifications were normalized to against *hprt* mRNA expression levels. **b** Isolated BMMs were stimulated with M-CSF for the indicated times, followed by lysis and western blotting using specific antibodies against Spi-C, c-Fms, and β-actin. Data are presented as the mean ± SD of three independent experiments. **p* < 0.05, ***p* < 0.005 vs. non-treated cells.

**
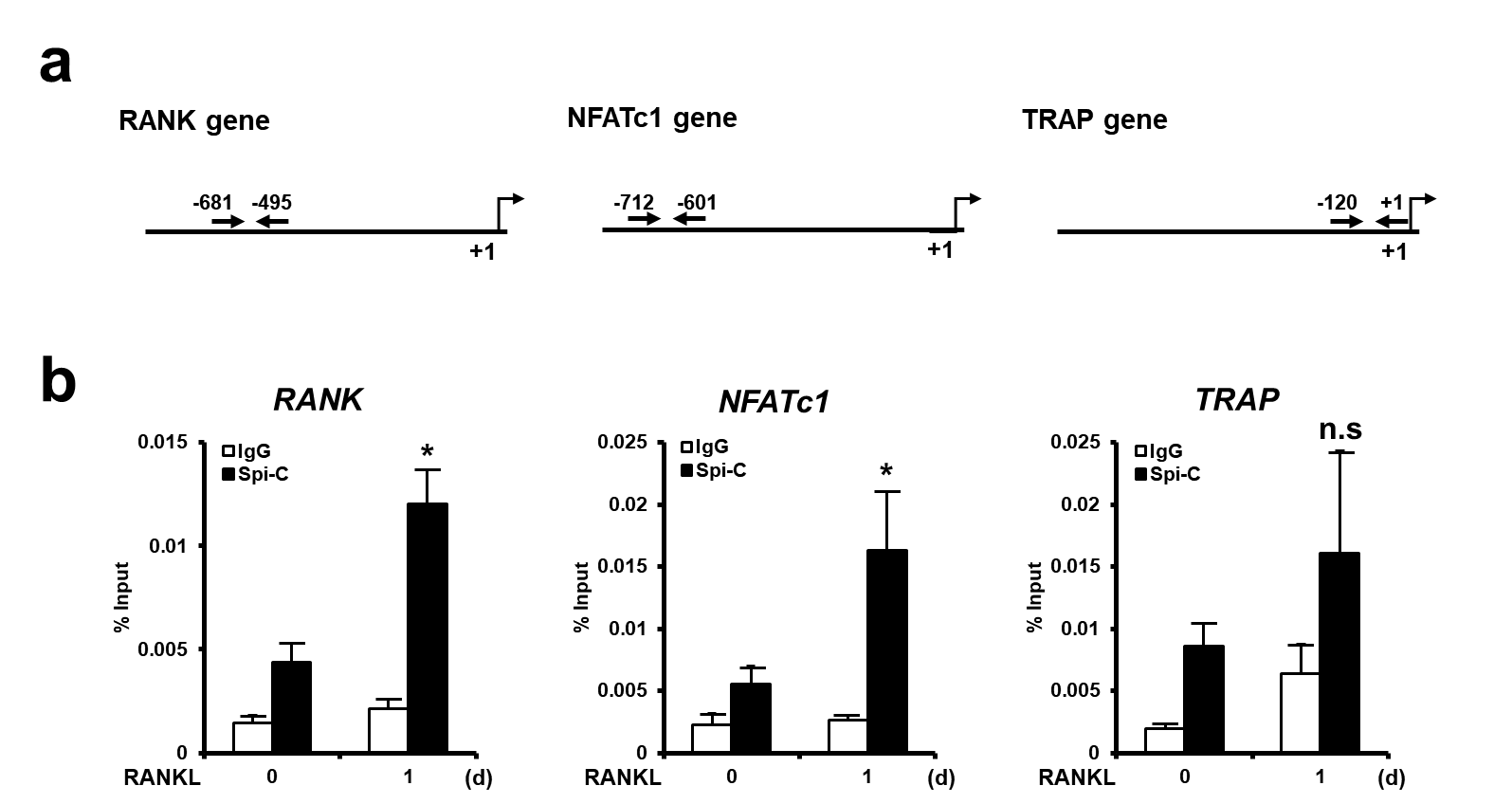
**

**Supplementary Figure S3. Spi-C binds to the *RANK* and *NFATc1* promoters. a** Schematic diagrams showing the *RNAK*, *NFATc1* and *TRAP* promoter regions, containing potential binding sites for Spi-C. **b** BMMs were treated with M-CSF (30 ng/ml) and RANKL (100 ng/ml) for 1 day; subsequently, ChIP assay was performed using Spi-C antibodies or IgG. Data are presented as the mean ± SD of three independent experiments. **p* < 0.05 vs. non-treated cells. n.s, not significant.

**Supplementary Methods**

**Reagents**

Recombinant human M-CSF was purchased from R&D Systems (Minneapolis, MN, USA). Antibodies against Spi-C and c-Fms were obtained from Santa Cruz Biotechnology Inc. (Santa Cruz, CA, USA).

**Isolation of mouse calvarial pre-osteoblasts and osteoblast differentiation**

Mouse calvarial pre-osteoblasts were obtained by excising the calvarial bone from 2-day-old neonatal mice, as described in Ref. 1^1^. For osteoblast differentiation, the cells were cultured for 7-14 days in an osteogenic medium containing 10 mM β-glycerol phosphate (Sigma), 50 μg/ml ascorbate-2-phosphate (Sigma), 10^-7^ M dexamethasone (Sigma), and 25 ng/ml human recombinant bone morphogenetic protein 2 (BMP-2, R&D systems).

**ChIP assay**

The ChIP assay was performed using the protocol recommended by Millipore. In brief, BMMs were cross-linked with 1% formaldehyde for 10 min at 37 °C. The cells were lysed in 200 μl lysis buffer and sonicated on ice (Branson Digital Sonifier) to shear DNA into pieces with an average size of 500 bp. After centrifugation, supernatants were pre-cleared with 50% salmon sperm DNA/protein A agarose slurry for 1 h. Chromatin was incubated with 2 μg normal IgG or anti-Spi-C antibodies, O/N with rotation. The sample were then incubated again with salmon sperm DNA/protein A agarose slurry for 1 h, followed by sequential washing with buffers. After elution, the complexes were reverse cross-linked with NaCl for 4 h at 65 °C and digested with proteinase K, EDTA, and Tris-HCl for 1 h at 45 °C. Purified DNA was subjected to quantitative real-time PCR with primers. Input DNA (1%) was used for normalization. Enrichment was measured using qPCR of immunoprecipitated DNA with following primers : RANK forward 5’-GGCACTCAAGGAATTGAAAAC-3’ and reverse 5’-TCATTACTGGCTTCTGGG-3’; NFATc1 forward 5’-GGGACGCCCATGCAATCTGT-3’ and reverse 5’-AAAATCGCAGGCTTCCCCCG-3’; TRAP forward 5’-TTGGGTAGCACAGCTTGTC-3’ and reverse 5’-AACAGGAAGGAAGTGGGGTG-3’.

**Supplementary Reference**

1 Rosen, C. J. *et al.* Circulating and skeletal insulin-like growth factor-I (IGF-I) concentrations in two inbred strains of mice with different bone mineral densities. *Bone* **21**, 217-223, doi:10.1016/s8756-3282(97)00143-9 (1997).
